# Supplementary material for: Content-rich biological network constructed by mining PubMed abstracts
Source: BMC Bioinformatics. 2004 Oct 8;5:147. doi: 10.1186/1471-2105-5-147 (PMC528731; doi:10.1186/1471-2105-5-147)
Supplement: Additional File 2 — The original results of the above study (non-essential files are deleted to keep the file size under the limit set by BMC bioinformatics). [file 1471-2105-5-147-S2.bz2 › chilibotAdditionalFile2/dip40/454ID10477718E10466/html/CCL11_CCR5.html]

 


 **CCL11** and **CCR5** 
  
Found 50 abstracts in PubMed, retrieved 40.  
 

 What does Google say? 
 PDF only 
| .edu only 

---

**Interactive relationship** (e.g. stimulation, inhibition, etc)

**Inhibitory relationship**- The CC chemokines RANTES, MIP 1alpha, MIP 1beta, and Eotaxin  [ **CCL11** ]  can suppress the replication of  **CCR5**  and CCR3 dependent viruses, while SDF 1 alpha beta suppresses that of CXCR4 dependent strains.  Ref: 10463536 Dev Biol Stand, 1999
- In human monocytes pretreatment with eotaxin  [ **CCL11** ]  decreased responsiveness to MCP 1, a selective ligand for CCR2, as well as to RANTES and MIP 1 beta, which bind to  **CCR5** .  Ref: 11264152 Blood, 2001
**Neutral relationship**- The results demonstrate that eotaxin  [ **CCL11** ]  is a  **CCR5**  agonist and a CCR2 antagonist.  Ref: 11264152 Blood, 2001
- Similar effects were obtained with transfected cells expressing CCR2 or  **CCR5** , but here a difference became apparent Eotaxin  [ **CCL11** ]  triggered  **CCR5**  at a concentration of 100 nM but not CCR2 even at 1 microM, suggesting an antagonistic effect on this receptor.  Ref: 11264152 Blood, 2001
- In agreement with this observation, eotaxin  [ **CCL11** ]  induced internalization of  **CCR5**  but not of CCR2 in human monocytes and transfected cells.  Ref: 11264152 Blood, 2001
- Eotaxin  [ **CCL11** ]  is a natural antagonist for CCR2 and an agonist for  **CCR5** .  Ref: 11264152 Blood, 2001
- Increased responsiveness of murine eosinophils to MIP 1beta CCL4 and TCA 3 CCL1  [ **CCL11** ]  is mediated by their specific receptors,  **CCR5**  and CCR8.  Ref: 12050188 J Leukoc Biol, 2002

**Non-interactive relationship** (e.g. studied together, co-existance, homology, etc.)

- Immunohistochemistry for MIP 1beta, MIP 1alpha, RANTES, eotaxin  [ **CCL11** ] , and MCP 3 ligands for  **CCR5**  and or CCR3 revealed the presence of MIP 1beta predominantly in a subpopulation of reactive astrocytes, which were more widespread in AD than control brains, and MIP 1alpha predominantly in neurons and weakly in some microglia in both AD and controls.  Ref: 9665462 Am J Pathol, 1998
- ... macrophage inflammatory protein MIP 1alpha, MIP 1beta, MCP 1, and stromal cell derived factor 1 were inactive, suggesting a role for the eotaxin  [ **CCL11** ]  receptor, CCR3, and ruling out the involvement of CCR1, CCR2,  **CCR5** , and CXCR4.  Ref: 9687538 J Exp Med, 1998
- The most active chemokines for resting Th IL 10, in terms of calcium mobilization and in vitro migration, were in order of potency CCL2 monocyte chemoattractant protein 1, CCR2 ligand, CCL4 macrophage inflammatory protein 1beta,  **CCR5**  ligand, CCL3 macrophage inflammatory protein 1alpha, CCR1 5 ligand, CCL17 thymus and activation regulated chemokine, CCR4 ligand, CCL1  [ **CCL11** ]  I 309, CCR8 ligand, CXCL12 stromal derived factor 1, CXCR4, and  **CCL11**  eotaxin  [ **CCL11** ] , CCR3 ligand.  Ref: 11145678 J Immunol, 2001
- This study shows that eotaxin  [ **CCL11** ]  also interacts with CCR2 and  **CCR5**  and can, thus, affect the responses of monocytes, which express both receptors.  Ref: 11264152 Blood, 2001
- Intracellular calcium levels in astrocytes were found to increase following application of RANTES, MIP 1beta  **CCR5**  agonist, SDF 1alpha CXCR4 agonist, but not eotaxin  [ **CCL11** ]  CCR3 agonist.  Ref: 11329179 Glia, 2001
